# MiRNAs and circRNAs for the Diagnosis of Anthracycline-Induced Cardiotoxicity in Breast Cancer Patients: A Narrative Review

**DOI:** 10.3390/jpm12071059

**Published:** 2022-06-28

**Authors:** Roberto Rosenfeld, Silvia Riondino, Vincenzo Formica, Francesco Torino, Eugenio Martuscelli, Mario Roselli

**Affiliations:** 1 Department of Systems Medicine, University of Rome Tor Vergata, Via Montpellier 1, 00133 Rome, Italy; roberto.rosenfeld88@gmail.com (R.R.); vincenzo.formica@uniroma2.it (V.F.); torino@med.uniroma2.it (F.T.); 2 Department of Internal Medicine, University of Rome Tor Vergata, Via Montpellier 1, 00133 Rome, Italy; eugenio.martuscelli@uniroma2.it

**Keywords:** mi-RNA, cardiotoxicity, anthracyclines

## Abstract

Breast cancer (BC) is the most frequent type of female cancer with increasing incidence in recent years. Doxorubicin (DOX) is an important backbone chemotherapy in BC, responsible for cardiotoxicity (CTX) in about 9% of treated women within the first year. Biomarkers of early CTX diagnosis are essential to avoid complicated DOX-related cardiac diseases. Traditional serum biomarkers are either poorly sensitive with transient elevation, and even absent if investigated outside their diagnostic window, or arise only in late-stage CTX. Emerging biomarkers such as non-coding RNA (ncRNA) have been recently investigated in DOX-related CTX. In our review, we revised the role of microRNAs, the most studied type of ncRNA, both in animal and human models, highlighting the interesting but often contrasting results. Moreover, we reviewed a novel class of ncRNA, circular RNA (circRNA), focusing on their modulatory mechanisms also involving microRNAs. MicroRNA and circRNA are players in a wide homeostatic balance with their perturbation representing a possible compensation for DOX damage. Further studies are required to assess the modalities of early detection of their variation in BC patients suffering from heart disease induced by DOX treatment.

## 1. Introduction

Breast cancer (BC) is the commonest female cancer worldwide, accounting for about 30% of female cancers, with a mortality-to-incidence ratio of 15% [1]. Globally, in 2020, about 19.3 million women were estimated to be diagnosed with BC and about 685,000 women died due to this malignancy [2]. Notably, it was estimated that in 2021 approximately 7.8 million women diagnosed with BC were alive due to early diagnosis and increasingly effective treatments [2]. Currently, a multimodal approach represents the optimal treatment strategy for BC patients, including surgery, radiotherapy, chemotherapy, hormone therapy, and targeted therapies, according to the stage and cancer cell molecular signature [1]. Nonetheless, acute and long-lasting toxicities of anticancer treatments, including cardiotoxicity, remain important unresolved issues. Anthracyclines (Doxorubicin, DOX; Epirubicin, EPI) have a key role in therapeutic strategies for BC patients since they significantly improve both disease-free and overall survival [1]. Suggested mechanisms of action of anthracyclines include the inhibition of the topoisomerase II, DNA adductions, oxidative stress, calcium homeostatic alterations, and immune response regulation [3]. Despite their efficacy, anthracyclines may lead to a variable rate of cardiotoxicity (CTX); therefore, a careful evaluation of features that might increase the risk of cardiotoxicity (age, neoadjuvant vs. adjuvant chemotherapy setting, methods of drug administration, prior cardiac events, family history of coronary artery disease, hypertension, and smoking habit) should be performed before the treatment choice [4]. As evidenced in milestone studies, anthracyclines induce cardiac damage with acute, sub-acute, and chronic patterns and a global incidence of about 9% [5] highlighting the urge for tools capable of detecting signs of cardiac suffering. About 95% of cardiotoxicities occur in the first year post-treatment [5] through mechanisms involving inhibition of Top-2a, oxidative stress, cell necrosis, and immunogenicity [3]. CTX was recently defined by the European Society of Cardiology (ESC) and the European Association of Cardiovascular Imaging (EACVI) as the fall of the left ventricular ejection fraction (LVEF) below the absolute value of 50% or the decline from baseline levels of 10% below the value of 54% for women and 52% for men. The decrease in the absolute value of the global longitudinal strain (GLS) of −18% or its decrease of 15% from baselines and, more generally, a relevant newly diagnosed cardiac pathology such as myocardial infarction (MI), heart failure (HF), and fatal arrhythmias are also considered [6]. Classically, the biomarkers used for identifying CTX during DOX treatment are cardiac troponins (cTn) isoforms I and T natriuretic peptides (NPs) [7], although N-terminal proB-type natriuretic peptide (NT-pro-BNP) is significantly associated with the presence of HF particularly in the late-stage DOX-induced cardiac impairment [8]. Currently, the most useful prognosticator of cardiotoxicity during chemotherapy is the Speckle tracking echocardiography (STE) that evaluates myocardial deformation as a marker of contractility and elasticity [9]. 

Additional tools for early identification of cardiac injury are under investigation. New approaches are oriented towards the study of microRNAs (miRNAs). MiRNAs are small non-coding RNAs consisting of 19–23 nucleotides, part of the larger group of noncoding RNA (ncRNA) [10]. They actively participate to embryonic and postnatal development [11] and other physio-pathological conditions and act as post-transcriptional inhibitors of mRNA through two main mechanisms that could be summarized as follows: (i) a complete bond with the target mRNA that leads to the degradation of the complex miRNA-mRNA, (ii) an incomplete bond of the miRNA-mRNA complex that is not degraded but remains sequestered; in either way, the final transcription of the target mRNA is hampered [10]. Apart from regulating intracellular processes, miRNAs are also delivered in the blood by exosomes, small extracellular vesicles that abound in the circulation to facilitate cell-to-cell communication. Exosomal miRNAs are found in many body fluids and are proposed as biomarkers for early and non-invasive cancer diagnosis by means of liquid biopsy [12]. Exosomes represent a rising area of interest as they carry key miRNAs involved in CTX [13,14].

NcRNAs include, among others, also circular RNAs (circRNAs) [15,16] and long non-coding RNAs (lncRNAs) [17] that can be aberrantly expressed in various diseases. CircRNA are molecules produced as covalently closed continuous loops that lack 3′- and 5′-ends and, therefore, have a higher tolerance to degradation by RNase R exonuclease [15]. They can be classified based on their origin as exonic circRNA (ecircRNA), if they are produced by exon splicing, circular intronic RNA (ciRNA), if the molecule derives from an intronic region splicing, exon-intron circRNA (EIciRNA), if the splicing process regards both exons and introns [16].

Several studies were directed towards the role of ncRNA in response to anticancer therapies [18,19]. Murine models were particularly versatile for studying dysregulation of ncRNA in the heart tissue and for comparing results with human cardiac cell lines. More recent studies focused on the detection and quantification of these molecules in human body fluids, such as plasma or serum.

The present review aims at providing an updated analysis on the diagnostic value of miRNAs, circRNAs, and in the setting of early anthracycline-induced CTX. 

We examined study methods, CTX definitions, and differences between animal and human models. Possible protective markers were also explored.

## 2. Role of microRNAs

### 2.1. Role of miRNAs Investigated in the Heart Tissues of Experimental Models

**Figure 1:**
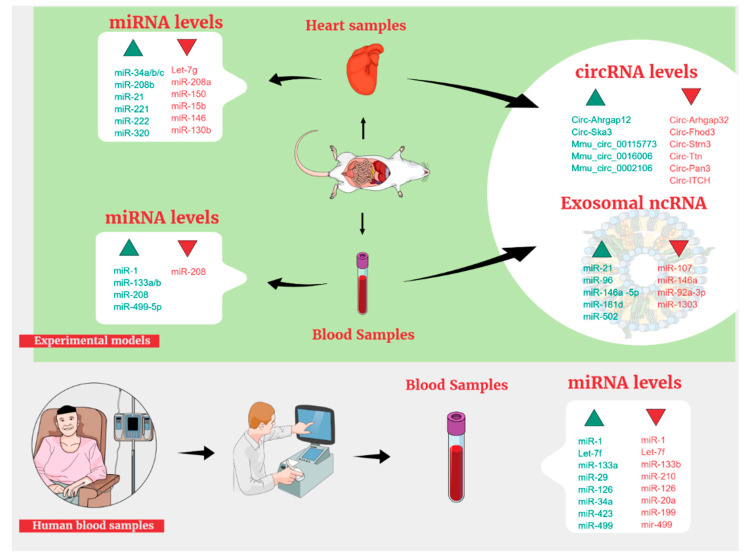
ncRNA and exosomes known as predictors of cardiac damage.

In the past ten years, a large number of studies were performed appraising the diagnostic value of miRNAs in different experimental models, both in vivo and in vitro. Their differential expression can have peculiar associations with the onset of cardiotoxicity (Figure 1). Moreover, the variation in their concentrations might have a protective or deleterious value. DOX remains the most cardiotoxic drug and, for this reason, vastly studied in this area. Among topoisomerase II inhibitors, DOX but not Etoposide, administered at different cumulative doses, was found to induce several types of miRNA modifications. It is worth underlying that Dexrazoxane (DZR) is used as a preventive agent of DOX-induced cardiotoxicity protecting cardiomyocites from oxidative stress. Notably, DZR was found to modulate miR-17-5p that seems to have a role in preventing DOX-induced apoptosis [20,21]. In an in vivo study on rats, treatment with the highest cumulative dose of Doxorubicin of 12 mg/kg (3 mg/kg for 4 weeks) resulted in the increase of 17 miRNAs and in the decrease of 8 miRNAs from their basal values [22]. Among them, upregulation of miR-34c, miR-208b, miR-216b, and miR-367 was positively correlated with the severity of cardiac tissue lesions (Table 1).

However, MiR-216b was found to be increased also at the minimal dose of 1 mg/kg per week for 2 weeks in the absence of cardiac abnormalities, probably as a consequence of a para-physiological drug-induced stress-response. Conversely, miR-216b and miR-367 increased in rats treated with DOX doses of 2 and 3 mg/kg for 2 weeks, and such an increase paralleled dose-dependent and dose-cumulative heart damage, suggesting their potential role as early biomarkers [22]. Moreover, also miR-34a proved trustworthy as an early marker [23]; indeed, in male B6C3F mice, DOX administered at the dose of 3 mg/kg weekly for 2–8 weeks (Table 1) reached progressive cumulative doses in different groups [23]. Eventually, the mice were sacrificed a week after the last infusion trying to mime a clinical late CTX. In this case, the hypertrophy-related miR-150 was found to be downregulated, whereas miR-34 and miR-208 were increased at high cumulative doses. However, also miR-34a was increased precociously, at all concentrations, and dose-dependently, supporting its reliability in early detection phases [23]. The role of miR-34 was also highlighted by Piegari et al. who treated rat cardiac stem cells (CSC) with an in vitro inhibitor of miR-34c 24 h prior to DOX administration, maintaining CSC vitality and proliferative potential with a significant reduction in CTX [28]. CSC lines were also used by Holmgren et al. to describe anthracycline-induced early and late abnormalities associated with an altered expression of miR-15b, miR-34a, miR-34b, miR-130a, miR-146a, miR-187, miR-199a, miR-199b, miR-214, and miR-424 during and after the treatment [30]. These interesting results are peculiar, but they could be attributable only to stem cell lines; in fact, in the literature, different miRNAs were observed to be involved in vivo. The hypertrophy-inducer miRNA, miR-21, was investigated in mice for its anti-apoptotic role by Tong et al. in acute and chronic DOX-induced CTX settings [25]. Surprisingly, miR-21 expression was depressed only in the chronic setting along with a significant heart index (heart weight/mice weight) decrease, while in acute CTX, it did not show any significant different expression. In the same research, mice with chronic heart injury had an absolute 20% survival reduction compared to those with acute injury, suggesting a possible protective role that was lost over time. Indeed, when miR-21 was transfected in mice, a reduction of 18–37% of cell apoptosis was attained, whereas the death cell increased by 57% when mir-21 antagonist was transfected [25]. This result led the authors to hypothesize that a probable target of miR-21 was the BTG2 gene, which is involved in proliferation pathways in myocardial cell lines that could be inhibited by the chronic exposure to DOX [25]. Beside miR-21, other hypertrophy-related miRNAs (miR-221, miR-222, and miR-208b) are found to be over-expressed only at high doses of DOX, and this suggests a sort of activation threshold [32]. Probably, the upregulation of this cluster begins as a protective regenerative stimulus but becomes predictive for cardiac damage in association with the increasing levels in cardiac Troponin T (cTnT) at the DOX dose of 18mg/kg and with the appearance of intracellular vacuolations at the cumulative dose of 24 mg/kg, confirming to be very tardive manifestations [23]. MiR-208, a miRNA involved in cardiac fibrosis and in hypertrophy, was investigated for causing CTX in murine models by Tony et al. with three groups of mice treated at different doses (Table 1) versus the combination DOX + miR-208a-antagomir group [26]. In this study, DOX levels increased miR-208 levels four-fold while the presence of antagomir resulted in an important reduction in mortality (47.8% DOX vs. 12.5% DOX + miR-208a-antagomir), although this result did not reach statistical significance. The pathological involvement of miR-208a was confirmed showing the post-transcriptional inhibition of GATA4 as the miR-208 target [26]. Novak et al. assessed miR-208 and let-7g tissue levels for the first time in rats treated with liposomal-DOX comparing the results obtained with DOX treatment or placebo [31]. The authors reported a decrease in miR-208 in the left ventricle, in contrast with previous studies which described an elevation [22,23,26,32], whereas let-7g was mostly decreased in atria bilaterally in agreement with previous experiments [24]. As expected, liposomal-DOX triggered a less marked reduction in miR-208a in the left ventricle (38.9% DOX vs. 23.6% L-DOX, respectively), confirming it to be a less stressful anthracycline. Unfortunately, cardiac assessments were not performed; therefore, it was not possible to ascertain if these results were associated with CTX nor to define their early release. In a model of acute toxicity carried out on cardiomyocytes of rats treated with escalated DOX daily doses of 3 mg/kg, Let-7g was found to be decreased, along with a significant increase in cTnT above the dose of 18 mg/kg [24], similarly to what later was observed by Desai et al. [23]. The acute toxicity setting was further explored in C57BL/6 female mice cardiac tissue upon DOX treatment (24 mg/kg cumulative dose for 2 weeks) in vivo [33]. A strong association with DOX-induced cardiotoxicity was observed only for miR-34a-5p and miR-451a, but what appeared more interesting was the altered expression of diverse miRNAs observed in the different separated cardiac chambers [33]. The implication of these results is that different miRNAs are expressed upon activation of distinct pathways leading to dysfunction onset. MiR-320 is a marker that emerged from recent studies investigating atherogenesis on animal models and in human settings [29]. Yin et al. reported a four-fold elevation of this biomarker in the cardiac tissue of mice that developed CTX and proved a causality between the increasing concentration of mir-320 and the depressed heart function echocardiographically investigated [29]. Indeed, upregulation of miR-320 worsened cardiac dysfunction and endothelial injury through targeting Vascular Endothelial Growth Factor A (VEGF-A), while inhibition of miR-320a reversed DOX-induced cardiac damage. However, the effects of miR-320 were not investigated on healthy mice not exposed to the anthracycline; thus, the results were somehow undermined [29]. Additional studies on miRNAs were explored with the purpose of elaborating a CTX-related profile. Among them, an intriguing experimental model induced cardiac damage and HF mechanically by left anterior descendant (LAD) coronary ligation with the aim to compare the results with DOX-induced cardiopathies [27]. In both groups of mice, the down-regulation of miR-29c, miR-30d, miR-30e, miR-133b, miR-143, miR-210, and miR-345-5p was equally observed, while they remained unmodified in a healthy control group (treated with a vehicle). However, following acute and sustained DOX treatment, only three members of the miR-30 family (in particular, miR-30a, miR-30d, and miR-30e) were found to be positively correlated with CTX [27].

### 2.2. Free Circulating miRNAs in Animal Models

Experimental models can provide in vivo information about circulating miRNAs and permit an easy way to perform dose-finding studies and intervening on administration timing, doses, or the number of cycles, which would be unethical on humans (Figure 1). In rats, studies on circulating miRNAs were largely heterogeneous, due to the use of several toxicants often different from DOX, use of different experimental models, and different kits used to analyze plasma samples, making any comparisons quite hard to implement. Mir-208, previously presented as a tissue biomarker, was also studied in rat plasma samples by Nishimura and colleagues [34] who, after an initial screening of 55 miRNAs, chose 7 miRNAs specific for heart tissue, which were present at higher concentrations in skeletal muscle. Among them, only miR-208 showed an expressing ratio higher than 10%. Unfortunately, these results were positively correlated only to isoproterenol (ISO) treatment, but not to DOX. Regrettably, the authors investigated only ISO for the chronic damage in a second step of the experiment, assuming a role only for this drug. However, different mechanisms are involved in acute and chronic damage, and DOX could have shown significant results in the chronic setting. Apart from speculations, two recent studies described BC patients experiencing CTX with an elevation of cardiac Troponin I (cTnI) and a decrease in LVEF, whereas no change in miR-208 was observed [35,36]. Therefore, the results of Nishimura et al. were considered not portable from rodents to human beings. In a different study, miR-208 together with miR-1 and miR-133b were explored as potential markers of both cardiac and skeletal muscle toxicity and were measured in the plasma of male Sprague Dawley rats treated with different toxicants chosen from the literature evidence, among which were isoproterenol (ISO), metaproterenol (MET), and allylamine (AAM) [37].

The results demonstrated that all three compounds caused cardiac and skeletal muscle injuries. However, a different behavior of the miRNAs was registered. In detail, both muscle and heart toxicities were strongly correlated with the increased miR-133a/b in serum, while an increase in miR-208 occurred only in cardiac toxicity. Finally, miR-1 was only slightly increased in both toxic conditions [37]. Therefore, the authors concluded that miR-208 could be used in combination with miR-133a/b to discriminate between cardiac and skeletal muscle toxicity [37]. Similar results were achieved by Liu et al., in a study in 2014, where miR-1, miR-133a, miR-208a, and mir-499-5p were investigated in superoxide dismutase-2 (Sod2+/−) mice. The authors recognized miR-208a as a relevant cardiac circulating biomarker given the significant plasma levels’ elevation, together with miR-499-5p which proved to be significantly upregulated, although to a lesser extent, following isoproterenol injection [38]. Noteworthy, in the same study, cardiac tissue evaluation indicated that miR-208a was decreased in the ventricles but not in atria, suggesting a tissue specific mechanism. Moreover, a disagreement about miR-208b differential expression could be observed between plasma upregulated levels and tissue downregulated levels. Further, a correlation among miR-208b, cTnI elevation, and cardiac degeneration, such as fibrosis and inflammatory infiltrating cells, became evident in 6 out 10 mice at the dose of 80 mg/kg and in all mice treated with the dose of 160 mg/kg of ISO [38]. MiR-1 and miR-133a/b were also evaluated in female mice in a direct comparison study between the effects of DOX and Imatinib mesylate (a tyrosine kinase inhibitor directed to the c-Kit mutation) [39]. Interestingly, miR-1, miR-133a/b were upregulated in both groups, while DOX caused an exclusive elevation of miR-34a, and Imatinib led to a peculiar upregulation of miR-339, miR-193a, and miR-186. Importantly, Imatinib raised cTnT levels less markedly than DOX but still significantly [39].

### 2.3. Role of CircularRNA

CircRNA molecules belong to a recently discovered class of ncRNA, and, consequently, subjects of pioneering studies on DOX-induced CTX. CircRNAs were investigated as transcription modulators, and very interesting results were achieved studying their role as competing endogenous RNAs (ceRNAs) [40]. In particular, circRNAs absolve their regulatory function by sponging miRNAs and impeding their regular activity or association with RNA-binding proteins (RBPs), resulting in different effects, such as transcriptional silencing, signal translation modulation, and/or specific mRNAs degradation [40]. Indeed, in previous studies on cardiac pathologies, circRNAs emerged as having an opposite function, being alternatively both protective and risk factors. For instance, the circular RNA HRCR was reported to protect from hypertrophy and HF [41], whereas CircNCX1 was found to be responsible for causing myocardial ischaemic injury [42]. However, specific studies described peculiar patterns of dysregulation in anticancer therapies. A recent study investigated the role as the biomarker of circPAN3 in male C57BL/6J mice treated twice per week with 10 mg/kg DOX (cumulative dose of 20 mg/kg of DOX) and compared the results with a control group treated for a week with saline (Table 2) [43].

It was found that the decreasing concentration of the regulatory protein Quaking 5 (QKI5), a RBP relevant for circPAN3 maturation, together with the downregulation of circPAN3, was strongly related to DOX-induced CTX [43]. Moreover, by transfecting miR-31-5p-mimic molecules or miR-31-5p-antagomiR, they showed that QKI was a possible target of miR-31-5p after DOX cardiac injury, and an axis miR-31-5p/QKI/circPAN3 was thus postulated. In support of this hypothesis, a week after DOX intraperitoneal injection, miR-31-5p levels doubled while circ-PAN3 had halved [43]. Unfortunately, troponins were not dosed; thus, the study lacks important diagnostic information for considering these miRNAs an alternative assessment tool. Similarly, Gupta et al. studied the effect that the QKI family can produce both in vivo and in vitro. They administered DOX at 5 mg/kg intraperitoneally once a week for 5 weeks, and after a further week, the mice were euthanized (Table 2) and hearts explanted. A total of 5 RBP were evaluated for analysis; 4 of them (Cirbp, Mkrn1, Rbm3, and Thumpd1) were found to be upregulated, whereas QKI was downregulated [44]. Of note, decreased protein and mRNA levels of all Quaking isoforms (QKI5, QKI6, and QKI7) were found at the same time. Afterward, they validated 4 circular RNAs targeted by QKI5, and they performed functional analysis establishing that Ttn, Fhod3, and Strn3were upregulated, while Arhgap32 was negatively regulated [44]. Apparently, contrasting results for circArhgap12 were observed in another study, in mouse heart tissues of male C57BL/6J treated with 5 mg/kg DOX per week for 5 weeks. The authors showed that circArhgap12 increased after DOX administration, finding its maximum concentration in the cytoplasm where it sponged miR-135-5p, an important inducer of apoptosis and oxidative stress [46]. Using silence interfering RNA (si-circArhgap12) and miR-135a-5p-antagomir on primary rat cardiomyocytes, it was concluded that circArhgap12 protects from CTX by balancing miR-135a-5p. The latter, if overexpressed or if strongly inhibited, led to CTX probably via the ADCY1 pathway as suggested by the authors [46]. One possible explanation of the results in clear contrast to those of Gupta’s could lie in the fact that in the first study, the animals were sacrificed shortly after the last DOX injection [46], while in the other after a week [44]. In this case, we might speculate that circArhgap12 overexpression might represent an initial acute protective response to DOX toxicity that in times could shift in a downregulation. Of note, the QKI family was also studied as key regulators by Yan et al. who reported a study on C57 male mice divided in three experimental groups: a DOX-treated group inoculated with DOX 5mg/kg for 5 weeks, a group that received pre-treatment with Salidroxide (SAL), an antioxidant drug, for 2 weeks and then DOX with the same schedule (DOX+SAL), and a control group treated only with the vehicle. Echocardiographic exams were performed to assess cardiac damage one week after the last injection, and no statistical differences were reported between controls and DOX+SAL groups, suggesting a protective role of SAL against CTX [48]. The authors showed that the circular RNA, FoxO1, increased in DOX-treated mice, whereas QKI family proteins were downregulated, suggesting that Quaking could be a possible modulator of FoxO1. In this setting, FoxO1 disinhibition could lead to myocardial fibrosis and hypertrophy [48]. However, the applicability of these results to CTX is limited, because troponins were not dosed, and heart disease occurrence was not reported. Li et al., in 2021, investigated the changing in the plasma level of circSKA3 after DOX treatment with crescent concentrations (2.5 M, 5 M, and 10 μM) for 24 h in human cardiac AC16 cells [15]. They observed high expression of Circ-SKA3, while miR-1303 expression decreased concomitantly in a concentration-dependent manner, probably via circSKA3 sponging activity. Notably, TLR-4 (an inducer of the NF-Kb pathway and immune response activation pathway) was found increased as a target in the axis circSKA3/miR-1303/TLR, while circ-SKA3 knockdown attenuated DOX-induced apoptosis and increased cell viability [15]. Unfortunately, in vivo studies were not performed to confirm these results. Further studies on the molecular mechanisms for DOX-induced CTX come from evaluation of differentially expressed circRNA and mRNA profiles in a cardiac toxicity model in which male C57 mice were treated with DOX 15 mg/kg (Table 2) [47]. After preliminary analyses, 3 types of circRNA were found upregulated and validated: mmu_circ_0015773, mmu_circ_0002106, and mmu_circ_0016006. Of note, after an extensive link analysis with several possible target miRNAs, mmu_circ_0002106 proved capable to regulate mRNA expression via sponging miR-344g-3p and miR-22-3p, the latter being responsible in particular for cell growth, apoptosis, motility, and the cell cycle other than cardiovascular events. Indeed, lower levels of miR-22-3p were proved to be associated with worsening of myocardial injury, while higher levels of circ_0002106 increased cardiomyocyte loss. Moreover, mmu_circ_0015773 is implicated in the regulation of cell proliferation, the cell cycle, and cell apoptosis through four miRNAs (miR-470-5p, miR-679-5p, miR-296-3p, and miR-876-5p) [47]. Eventually, mmu_circ_0016006, involved principally in cell apoptosis, was recognized to be linked with five miRNAs, although only three of them were reported due to a consistent background in the literature (miR-466i-5p, miR-665-3p, and miR-466m-3p) [47]. In a study on human-induced pluripotent stem cell-derived cardiomyocytes (hiPSC-CMs) obtained from 6 human hearts explanted after autopsies, of which 4 were affected by structural disease and HF, molecular signs of cellular and mitochondrial oxidative stress, DNA damage markers together with cells’ contractile function (contraction velocity and deformation distance,) and the relaxation function (relaxation velocity and deformation distance) were used to define cardiac toxicities [45]. Among the 15 candidate miRNAs in hiPSC-CMs, only miR-330-5p was significantly downregulated when tumor-suppressive circular RNA, circITCH (circular RNA ITCH (E3 ubiquitin-protein ligase)), was overexpressed, identifying miR-330-5p as a potential target. Indeed, CTX in hiPSC-CMs was found to be associated with a significant underexpression of circITCH and with the upregulation of miR-330-5p. Interestingly, miR-330-5p sponging resulted in an improvement in contractile function through the upregulation of SIRT6, Survivin, and SERCA2a [45]. 

In conclusion, it seems reasonable that miRNAs expression profiles should be studied together with their strictly related modulators, such as circRNA and RBP, to define the landscape of the transcriptome better.

## 3. Studies Conducted on Exosomes in Experimental Models

### Exosomal Transportation for circRNA and miRNA in DOX-Induced Cardiotoxicity

Exosomes are nanosized components of 30 to 150 nm belonging to the family of Extracellular Vesicles (EV) together with apoptosomes and microvesicles derived from a process of endocytosis of the endosomal membrane [49]. They contain various bioactive molecules (Table 3), such as proteins or nucleic acids (mirNA, circRNAs, ecc), and can convoy a wide range of messages through ribonucleic molecules (usually coding RNA and ncRNA) to target cells in local environments or distant sites through a process of docking and target membrane-fusion to the cytomembrane of the cell destination.

Intercellular communication mediated by exosomes has an important role in modulating the physiological processes and pathological mechanisms [49]. As evidenced by Beaumier et al. in a study on cancer canine patients, there was a peculiar differential expression of miRNAs after administration of DOX [13]. Exosomes’ content was searched for dysregulated miRNAs (exo-miRNAs), and miR-107 and miR-146a were found downregulated, while miR-502 was increased starting from the second dose of DOX [13]. Cardiac damage assessment was performed by echocardiography, cTnI level measurement, and histological examination of a biopsy sample, although a LVEF decrease >10% was positively correlated with upregulation of miR-181d after completing the DOX treatment. Nonetheless, the incidence of echocardiographic alterations and overt heart disease or HF was low, and exo-miRNAs did not correlate with cardiac diseases or with cTnI elevation up to 1 month post-DOX; thus, a linking proof with early CTX was missing [13]. Exosomes derived from stem cells were proven to have a role in the treatment of DOX-induced cardiomyopathy. Studies on cardiac mesenchymal stem cells (cSMC) stimulated with the macrophage migration inhibitory factor (MIF) prior to DOX administration resulted in the formation of MIF-containing exosomes (Exo-MIF) in which lncRNA–NEAT1, suggested to exert a significant role in the cardioprotective process, accumulated [51]. The results demonstrated that Exo-MIF relieved Dox-induced cardiomyocyte senescence, and these cardioprotective properties were explained by lncRNA NEAT1 presence that regulates these pathways sponging miR-221-3p and had been already found to have protective roles against cell death, apoptosis, and cell senescence [57]. In particular, miR-221-3p upregulation, through the regulation of SIRT2, could lead the cells in the G0/G1 phase, increase the expression of p27 and p16, while, on the other side, inhibiting telomerase activity, leading the cell to senescence. Therefore, it was speculated that Exogenous Exo-MIF might increase the concentration of NEAT-1 that, in turn, could contrast DOX-induced cardiac damage antagonizing early senescence [51]. A similar investigation studied lnc-MALAT1, a hypoxia-inducible lncRNA, in preconditioned human cMSCs with hypoxic stimuli. The cells were treated with DOX 0.5 μM for 24 h, and exosomes were collected from hypoxic culture (Hypo-Exo) cell supernatant [54]. The results showed that lnc-MALAT-1 modulated miR-92a-3p, a miRNA responsible for triggering cell proliferation and senescence, which in turn negatively regulated ATG-4a, an important positive regulator of autophagy and cell self-renewal, leading to a pro-senescent stimulus (Regulatory Axis lnc-RNA-MALAT-1/miR-92a-3p/ATG4) [54]. Notably, Hypo-Exos protected DOX-treated cardiomyocytes from early senescence by avoiding a larger fraction of cells entering in the G0/G1 phase. The presence of lncRNA-MALAT1 and its over-expression in Hypo-Exo was considered as a probable response to hypoxic stressful stimulation [54]. The same authors suggested the cardioprotective potential of exosomes transporting miR-199a-3p in DOX induced senescent cardiomyocytes and senescence-associated secretory phenotype (SASP) cardiomyocytes [53]. They showed that miR-199a-3p was downregulated in cardiomyocytes when exposed to DOX, while its overexpression promoted cell proliferation, reducing cardiac senescence, and inhibited SASP generation through the inhibition of GATA-4 protein, the most important factor for programmed senescence and damage-induced senescence. Indeed, exosomes were harvested from DOX-treated cells (Exo-DOX) and induced early senescence when used on healthy cardiomyocytes, thus confirming the existence of SASP [53]. Analogously, Lee et al. in 2021 conducted research on miR-199a-3p with the aim to define the relationship with the Survivin protein via the Akt-Sp1/p53 pathway. For this reason, they used a cMSC cell line to harvest exosomes (cMSC-EV), used to pre-treat H9c2 cardiac myoblast cells, which consequently were exposed to DOX (1 µM, for 24 h) for in vitro studies [55]. Exosomes were isolated from murine embryonic mesenchymal progenitor cell culture media using ultrafiltration procedures, and echocardiography assessment was used to define CTX in mice. For in vivo studies, male C57BL/6 mice were inoculated with cMSC-sEVs intravenously, and consequently DOX infusion was administered (15 mg/kg, i.p.); mice were then sacrificed 14 days after DOX treatment. Three miRNAs (miR-199a-3p, miR-424-5p, and miR-21-5p) were studied in MSC-sEVs, but only miR-199a-3p caused Survivin and Bcl-2 upregulation. Furthermore, using cMSC-sEVs, the authors demonstrated a partial reversibility of the DOX-induced LVEF depression [55]. Regrettably, neither cTnI investigations nor a correlation study between CTX signs and miRNA levels were performed. In HER-2 positive neoplasms, the treatment includes the administration of Trastuzumab in association with DOX, and this might result in a further worsening of CTX. In a recent article, Milano et al. proposed an investigation on animal models on the combination of DOX and trastuzumab [50]. Exosomes were collected from human cardiac progenitor cells (Exo-CPC) and injected in adult female rats treated with DOX; indeed, Exo-CPC contained antioxidant proteins such as Super Oxyde Dismutase 2, thrombospondin 1, and collagen 1A1 and prevented CTX not only through antioxidant effects but also via miR-146a-5p overexpression. Indeed, Exo-CPC, by suppressing the expression of miR-146a-5p target genes (such as Traf6, Smad4, Irak1, Nox4, and Mpo, all signaling mediators of inflammatory and cell death axes), inhibited DOX/Trastuzumab-mediated oxidative stress in cardiomyocytes [50]. A study on Sprague Dawley mice treated with 6 cycles of DOX until the standard c.d. of 15 mg/kg demonstrated that injection of exosomes collected from bone marrow MSC (Exo-BMSC) had a protective effect on DOX-induced damage [56]. In particular, miR-96 was downregulated, while its target Rac1 was upregulated. At the same time, the nuclear factor-κB (NF-Kb) signaling pathway was found to be activated after DOX treatment, probably by the upregulated Rac-1 via the axis miR-96/Rac-1/NF-Kb [56]. These pieces of evidence provide a strong substrate to design ways of exosome content delivering to limit cardiac injury. Indeed, as the heart tissue seems to be refractory to absorb exosomes, an interesting research project proposed to use microbubbles to destroy exosomes causing the release of their content in selected cardiac regions [52]. The authors ultrasonographically documented a limitation of CTX as a result of miR-21 delivery, and demonstrated that cardiac function was significantly restored by the treatment with Exo-miR-21 [52]. In conclusion, exosomes have proven to be a surprising source of biomarkers for early diagnosis of CTX, providing also a protection for ribonucleotides from RNAs that results in their stabilization. Methodology, however, suffers several limitations (Table 3): procedures for isolation and purification require expertise, and tools have peculiar drawbacks; for instance ultracentrifugation, the gold standard method, is cumbersome and time-consuming, and the high rotations could break a part of the exosomes with consequent loss of material, while exosomes erroneously collected might undermine the researchers’ results [49].

## 4. Studies on Human Blood Samples

Nowadays, studies focus on human plasma samples to avoid obvious portability problems. A common problem of almost all studies based on samples collected from human patients is that BC patients are often treated with a combination of drugs with different CTX such as DOX administered together with cyclophosphamide (AC regimen) [58] or AC in combination with the sequential Paclitaxel [35], while DOX alone is rarely administered (Table 4).

Furthermore, observational studies have the persistent problem of selection biases because it is pivotal, when randomization is not an option, to exclude patients that are already on cardioprotective therapy (ACE-inhibitors and beta-blockers) in order to observe unmodified results (Table 4). In the literature, only Rigaud et al. excluded patients on cardioprotective treatment [35]. A recent systematic review tried to define a molecular signature evaluating a differential expression of miRNA in studies selected for BC patients who received cancer therapy with anthracyclines and experienced any form of CTX [66]. Among the initial 40 miRNAs considered, only five miRNAs (Let-7f, miR-1, miR-20a, miR-126, miR-210) recurred and presented a differential expression path after anthracycline-based therapy, having thus the potential to predict anthracycline-induced cardiotoxicity in BC patients [66]. The authors retrieved two Reactome pathways shared by multiple miRNAs, such as “Signal transduction R-HAS-162582” (sharing let-7f, miR-1, miR-20a, and miR-126), responsible of endothelial homeostasis, oxidative stress, apoptosis, cell growth, and angiogenesis, and the pathway of “Cellular responses to stress RHAS-2262752” (sharing let-7f, miR-20a, and miR-210), responsible for pathways involved in response to reactive oxygen species and cycle redox [66]. Therefore, these miRNAs not only indicate that different pathways lead to cardiotoxicity, but also that cell responses to stress and signal transduction pathways may contribute to such cardiotoxicity. A later small study identified and tested another panel of miRNAs (miR-126, miR-34a, miR-499, miR-29a, and miR-423) in BC patients followed-up for 6 months and correlated the changes of these markers with specific markers of cardiac injury, such as cardiac troponin I and T, to demonstrate their specificity [64]. The panel of miRNAs employed was chosen for their role in stimulating cardiac remodeling, cardiac vasculature damage, and, overall, in chemotherapy-induced cardiac dysfunction [28,35,36,59,67]. The study confirmed the role of all tested miRNAs in anthracycline-induced CTX and peculiar correlations with troponins: miR-29a, miR-34a, and miR-126 with cardiac troponin I, and miR-126, mIR-423, and miR-499 with cardiac troponin T [64]. MiR-1 is normally described, in elevated quantities, in cardiac tissue as a modulator of the interventricular septum trophism [68,69]. The miR-1 expression balance is pivotal for the correct growth of myocardial tissue, and its elevation is important for developing pressure-overload-induced cardiac hypertrophy, myocardial fibrosis, and dysregulation of calcium homeostasis [70]. After myocardial infarction (MI), miR-1 was also proven as a useful biomarker for cardiac remodeling and oxidative stress [71]. Indeed, it was found to be responsible for the aberrant expression of fetal cardiac genes such as Atrial Natriuretic Factor (Anf), skeletal muscle α-Actin (Acta1), cardiac alpha-Actinin (Actc1), as well as the contractile protein isoforms α- and β-Myosin Heavy chain (Myh6 and Myh7, respectively) [70]. In DOX-treated BC patients, miR-1 disclosed discordant results. Indeed, Rigaud et al. described for the first time an elevation in miR-1 plasma levels that paralleled the increasing plasma levels of cTnI [35], while Todorova et al. found that miR-1 was down-regulated after the first infusion of DOX-chemotherapy [58]. On the other hand, several other studies did not detect any statistically significant differences in miR-1 levels [36,63], although the results could have been influenced by different methods, definitions for CTX events, kits and procedures used to detect miR-1, and different DOX-based protocols. In a pilot study on children treated with AC, plasma miR-1 was significantly upregulated 6, 12, and 24 h post-AC compared with controls (children receiving non-cardiotoxic chemotherapy) [59]. Further studies should be performed to determine if miR-1 has its major role during “acute” heart damage, administering a single dose of anthracycline [58,59] showing its maximum elevation during the acute phase [36]. Mir-20a is part of a wider group, the miR-17/92 cluster, recognized to be an oncogene, acting as an epigenetic inducer of proliferation and neo-angiogenesis in estrogen-negative patients [72]. Studies highlighted its role in DOX-related CTX decreasing in the blood samples of BC patients, according to manifestation of cardiotoxic events [60,61]. Notably, Qin et al. showed that miR-20a and miR-17-5p were independent factors predictive for a lesser CTX using multivariate logistic regression; in addition, the ROC curve was performed and confirmed the good predictivity for CTX (AUC 0.842, 95% CI 0.778–0.906) [61]. MiR-29 was previously reported to be associated with cardiac remodeling and hypertrophy [64]. In a recent study conducted on USA BC patients, this miRNA was found to be upregulated and positively correlated with cTnI elevation [64]. In the pilot study of Leger, miR-29b together with miR-499 was upregulated in acute AC-induced cardiac injury and was associated with troponin concentrations post-AC. Moreover, after AC treatment, only miR-29b and miR-499 expression significantly correlated with cumulative AC dose, indicating that they might be useful in detecting subclinical cardiotoxicity [59]. MiR-34a was found to be associated with cardiomyocyte apoptosis, cardiac fibrosis, and oxidative stress [28]. This biomarker proved to be upregulated in a recent study conducted on BC patients treated with DOX at every time point, but a concomitant increase in cTn was not observed [63]. Similarly, two older studies showed an elevation of miR-34a although it was not strictly correlated with CTX events [36,64]. MiR-126 is an oncosuppressor involved in cellular growth and in VEGF pathways [73]. DOX-related toxicity provokes a downregulation of miR-126 levels in the blood as shown by two recent studies on BC patients [60,61]. Lakhani et al. observed a strong correlation between miR-126 and troponins, both isoforms I and T [64]. Furthermore, epirubicin but not DOX, administered within the FEC100 scheme with sequential Taxan (Paclitaxel or Docetaxel), caused a marked elevation in miR-126 that, however, was not associated with CTX signs [36]. Moreover, results from Todorova et al. failed to report a differential expression selectively in patients with CTX events [58]. The limitations of this study were a low number of patients, the fact that miR-126, troponins, and NT-pro-BNP were not directly compared, and lastly, the low rate of CTX injuries ultrasonographically detected. The MiR-133 family is an important modulator of cellular apoptosis, cell survival, and, as previously described, it represents one of the strongest correlations between murine models and humans. Indeed, very recently, in a study involving 6 BC patients undergoing DOX, miR-133a and miR-133b displayed a peculiar behavior, the former being decreased, while the latter increased in serum [65]. Conversely, other mi-RNAs evaluated, miR-15, miR-208a, and miR-499, failed to show a significant dysregulation as compared to baseline levels [65]. MiR-199a is a recent biomarker evaluated for HF and upregulated in pathological hypertrophic hearts [62,74]. Recently, it was investigated for its possible predictive power for anthracycline-induced heart injuries in BC patients and was revealed to be upregulated in the first 6 h following anthracycline injection [36], whereas it was downregulated in a recent study in children treated with anthracyclines [75]. This could be due to the different settings, and the early upregulation observed in the first 6 h could represent an initial compensative stress response. Mir-210 is an epigenetic modulator of the response to hypoxia, of VEGF-induced neoangiogenesis and endothelial cell migration for new aberrant capillary formation, its up-regulation being crucial for cell survival, migration, and differentiation [76]. Results on mir-210 are discordant. Indeed, its blood concentrations were found decreased in several studies in patients with CTX effects [60,61], whereas a previous study did not prove miR-210 to be predictive for CTX in adult BC patients [58]. In addition, in pediatric patients, despite a four-fold serum increase, mir-210 was not associated with changes in LVEF or to CTX signs [75]. Mir-423 was observed in several studies as an inducer of cardiomyocyte apoptosis and HF [35]. After anthracycline-induced damage, Frères et al. reported a marked elevation of this marker in patients who experienced LVEF reduction more than ten points under 53% [36], confirmed in a recent study, observing the elevation of this miRNA along with the increase in cTnT but not in cTnI [64]. MiR-499 is a protective factor against cellular apoptosis, and it is a potential biomarker for MI and end-stage HF [75]. Many recent studies found a direct correlation between its elevation and CTX incidence, observing the rising in cTn and a LVEF decrease at ultrasonography (US) [64,75]. Notably, Gioffrè et al. observed miR-499 elevation associated with an increase in cTn, but no sign of damage was found at the US [63], suggesting an initial protective role. Similarly, a previous study in children treated with DOX showed a marked elevation of miR-499 from baseline, but no significant difference was observed between controls (non-cardiotoxic chemotherapy) and the CTX group at any time point [59]. Let-7f is a pro-angiogenic miRNA and an important prognostic factor in ischemic stroke, acting at the level of the Transforming Growth Factor-β and VEGF pathways [31]. It was found to be possibly responsible for LVEF depression and dilatative cardiopathy [58], demonstrating its cardioprotective role by reducing endothelial dysfunction [58]. Qin et al. and Zhu et al., who observed lowered levels of let-7f after an anthracycline-based therapy, conducted their studies in China with interesting results about Asiatic ethnicity but which could suffer indirectness when compared with similar Western studies and, more generally, with Caucasian people. Notably, Zhu et al., after logistic regression, described let-7f as an independent prognostic factor for CTX development and demonstrated that the combined evaluation in let-7f and miR-126 had a high predictive value after ROC curve analysis (0.885 95% CI 0.818–0.952) with a sensitivity and a specificity of 88.9% and 8.7%, respectively [60]. Moreover, after a pathway analysis, the gene LIN28A was found to be a target of let-7f; in fact, this gene is known to induce heart hypertrophy in mice [77] and participates in several paths regulating growth, pluripotency, and oncogenesis [78,79]. As evidenced by Gioffrè and colleagues, Epirubicin and Doxorubicin do not share the same mechanism of action; in fact, Epirubicin does not alter in the same way the expression pattern of the analyzed miRNAs [63]. A recent study on human epidermal growth factor receptor-2-positive (HER-2+) BC patients treated with epirubicin/cyclophosphamide followed by docetaxel plus trastuzumab (EC-D+T) adjuvant chemotherapy demonstrated a positive correlation between cTnl and miR-130, a proposed marker for cardiac damage and reduced cardiac function that operates through downregulation of connexin-43 [80]. Several promising miRNAs evidenced in single studies were not confirmed by later research. For example, the signature composed of miR 122-5p and miR-17-5p induced by DOX treatment observed by Gioffré et al. [63] was not described in other studies, to our best knowledge, but further highlighted the difference between DOX- and EPI-dependent CTX.

The emerging use of bioinformatic and data mining in predictive medicine permits the analysis of a wide amount of data and allows the simultaneous evaluation of enriched databases. Yadi et al. used this method to analyze a total of 2978 miRNAs establishing a miRNA-gene interaction network to screen for those related to cardiotoxicity in BC patients [81]. The authors described a closed association with the onset of anthracycline-induced cardiotoxicity of miR-4638-3p and miR-1273g-3p [81], confirming the wideness and heterogeneity of this expanding field of biomarker research.

## 5. Discussion

Cardiovascular disease has become an important source of morbidity and mortality in cancer survivors treated with chemotherapy, target therapy, or immunotherapy. The old classification of cardiac damage recognized two different types of treatment-related cardiomyocyte injury, type I and type II [82]. The former was typically associated with Anthracyclines and with cardiac damage consisting of cellular necrosis or apoptosis, whereas the latter was considered to be the elective for toxicity mechanisms of biological therapies such as anti-VEGF and anti-Her2 agents [82]. Growing evidence indicates that early diagnosis could allow cardiac function preservation in 80% of the population [5]. Classically, the recognition of a CTX event relies on clinical evidence, radiological signs (echocardiography or Magnetic Resonance), or biomarkers’ alterations. Although clinical trials mainly registered myocardial dysfunction and HF, there is heterogeneity among cardiotoxic events. Recent guidelines consider a broader variety of cardiac diseases of increasing interest such as arrhythmias, valve dysfunction, coronary artery disease at any stage, arterial or venous thromboembolism, immune myocarditis, and pericarditis [83]. The CARDIOTOX trial recently updated the true CTX incidences with the commonest oncological therapies using the most recent definitions of cardiotoxicity according to international guidelines [84]. Following anthracycline administration, mild CTX, such as biomarker elevation in otherwise asymptomatic patients or LVEF dysfunction in absence of organic disease, was a relatively common finding (44%) [84]. Of note, the incidence of moderate and severe CTX was assessed to be 2.7% and 3.1%, respectively [84]. These results, as also hypothesized by the authors, could underestimate the real-world CTX incidence [5,85] since patients with HF and LVEF severely depressed at baseline were excluded from the trial. As far as the radiological signs are concerned, the gold standard remains Magnetic Resonance Imaging (MRI), with fibrosis and extracellular edema being early findings predictive of cardiac precocious injuries given as standardized, comparable, and repeatable information [6]. On the other hand, the large use of low-cost Ultrasonography (US) allows a fast bedside examination of cardiac damage and ventricular dysfunction. Recently, the EACVI guidelines suggested considering as normal a LVEF above 54%, whereas a CTX event should be suspected for a lowering of 10% under the value of 54% or even less than 10% when an absolute reduction of 15% from the baseline is observed [6]. Major drawbacks of US are the variability of information if performed by different operators, difficulties to distinguish cardiac damage from myocardial dysfunction, and the possible delay in evidencing clear findings of alteration. With regard to the latter problem, a new technique of great value is the “speckle tracking echocardiography”, which is based on the GLS as a marker of cardiac wall stiffness [9,86]. A relative reduction of 15% or absolute values inferior to -18% are recognized by the most authoritative guidelines as a reliable tool of early diagnosis of CTX, anticipating the appearance of cardiac fibrosis and cardiac remodeling [86]. Nevertheless, recommendations suggest associating radiological examinations with serum biomarkers for early diagnosis of cardiac injury [7,86]. Several serum biomarkers have been proposed, but presently only TnI, TnT, and NT-pro-BNP are recognized as useful for diagnosing CTX. Troponins seem to have an early role as markers of DOX-induced damage since they rise for acute and sub-acute cardiac injuries, whereas NT-pro-BNP has a tardive role as the HF biomarker since it increases for atrial volume overload. They could increase earlier than radiological imaging signs but have an unsolved limitation. First, troponin elevations are difficult to detect because the right timing for blood sampling is highly variable. The recent choice to increase sensitivity of Tn testing (hs-Tn) has inevitably led to a lower specificity [86]. The research for novel biomarkers for early detection of cardiotoxicity in BC patients receiving anthracyclines is of utmost importance, and attention is focused on miRNAs. miRNAs play a pivotal role both in physiological cardiac development and during the pathological processes (AMI, arrhythmias, cardiac hypertrophy, HF); therefore, circulating miRNA content might serve as an indicator of tissue injury during cardiovascular disease [87]. However, their concrete use in this field is still in an embryonic stage, mainly due to the lack of reproducibility of the methods employed. In anthracycline-induced CTX research, different animal settings and procedures are used; several miRNAs are evaluated; anthracyclines are administered at different doses and timings; heterogeneous experimental models (mice, rats, cells) are tested, and different model subtypes (e.g., “Sprague Dawley” vs. “Wistar” rats) are used. With the aim to validate preclinical results in patients, some of the promising miRNAs were studied both in animals and humans, but only a few of them proved to be dysregulated in both settings. A recent systematic review considered Let-7f, miR-1, miR-20a, miR-126, and miR-210 to have the highest potential to predict anthracycline-induced cardiotoxicity in BC patients [66]. Among them, the let-7 family of miRNAs was found to be decreased both in mouse (let-7g) and human (let-7f) hearts with a strong correlation with cardiac damage. Therefore, only let-7f was proved to be a good predictor of CTX, working as an independent prognostic factor and showing a diagnostic role together with miR-126 in the early determination of CTX in predicting the development of echographic alterations or HF [60]. MiR-133a/b was shown to be a promising biomarker capable of predicting CTX, as the first studies on mice described a significant elevation of the miRNA plasma levels, highlighting a direct correlation with CTX [27,39], and similar results were also observed with other drugs (Imatinib) [27]. Notably, these results were confirmed also in BC patients [65]. Results had to be taken with caution, since 4 out 6 women were concomitantly treated with anti-Her-2 therapy (Trastuzumab) [65]. Among other relevant miRNAs, miR-423, a well-known inducer of cardiomyocyte apoptosis and HF [35], was recently investigated for its possible role in early diagnosis of CTX, and good correlations with both a cTnI increase and ultrasound abnormalities were observed [64]. Moreover, miR-29’s role in pathological animal cardiac hypertrophy was confirmed in patients [64] with good subclinical cardiac damage predictivity, especially in association with miR-499 [59]. Promising results came from small sample studies employing different toxicants such as ISO, in which hypertrophy-induced miRNAs (miR-221, miR-222) or anti-apoptotic miRNAs (miR-1, miR-21) were described to be associated with early CTX onset. Despite the above, however, results on dysregulated miRNAs in patients are largely discordant (Table 4). Similarly, miR-34 was found altered both in mice and in humans, but the results were not fully comparable due to the different methods and CTX definitions and due to the observed low incidence of CTX (Table 1 and Table 2). Conversely, the encouraging evidence coming from miR-208b in mice treated with ISO [34,37] failed to be confirmed in BC women treated with DOX [36]. MiRNAs could be considered as a part of a complex network where they are regulated by time- and space-dependent mechanisms. Interfering events such as DOX-induced damage could perturb this equilibrium, and miRNAs could be either upregulated or downregulated, depending on the concentration and exposure to DOX, in an attempt to establish a new homeostasis. Indeed, as we observed in several studies, not always does an increased miRNA parallel that of cardiotoxic signs such as cTn elevation [36,63,64]. One of the major problems encountered in human studies is represented the heterogeneity of the adopted methods, of the target populations [59,75], and of the cardiotoxic oncological therapies used (doxorubicin, epirubicin, trastuzumab, but also Paclitaxel and Cyclophosphamide) [35,36,58,60,61]. This might have led to discordant results: for example, miR-126 was increased in some studies [36,58], while in other studies it was reduced [60,61]. When similar methods were employed, results were indeed more concordant [60,61]. A second problem is represented by the lack of correlation between assessed clinical CTX and biomarker reduction (Table 4), as observed in the lack of correlation between miR-1 or miR-34 and detected LVEF abnormality [58]. Analogously, miR was increased along with hs-cTnT in children exposed to anthracyclines, but echocardiographic abnormalities were not significant [59]. Similar discordant results were described for miR-210 [58,60,61,75]. MiRNAs are not the solely investigated predictors of CTX, and interesting new branches regarding circRNA are emerging. In the investigational area of DOX-induced cardiotoxicity, circRNAs are also known as competing endogenous RNAs as they compete in some cardiac functions with miRNA by sponging them and creating a second level of regulation [41]. Several investigations were performed to ascertain their role in DOX-induced CTX and their possible use as biomarkers (Table 3), many of them with discordant results possibly due, again, to different experimental conditions [44,46]. These studies, however, are important to clarify mechanisms of pathways of a wider background, suggesting that after DOX treatment, miRNAs’ dysregulation could have a second level of pathophysiological modulation [46]. These pieces of evidence indicate that efforts should focus on the better understanding of the biological processes underlying miRNA release, modulation, and adaptive and even protective responses to specific cardiac injuries.

## 6. Conclusions

Circulating biomarkers, including microRNAs, might uncover early signs of heart injury before overt cardiovascular damage is established. However, the significance of biomarker fluctuations must be carefully framed in the context of the right pathological scenario. MiRNA dysregulation, in fact, could represent an adaptive stress response and a continuum with the late, consecutive, pathologic hypertrophic stimulation and HF. The challenge of the diagnostic role of miRNA is to determine when a miRNA elevation stops to be beneficial and starts to be a marker of damage or even noxious effects. Different methods and different timings of sample collection have been used so far, and the differences in these experimental approaches have caused problems of reproducibility and portability of animal studies on humans, with the inevitable heterogeneity and the consequent enormous disadvantage of providing dispersive results. On the other hand, they have been useful for building a panorama for each type of miRNA in terms of acute and chronic conditions. In this light, future perspective studies cannot disregard the common definitions of cardiotoxicity, experimental models, and inclusion/exclusion criteria to ensure a basilar homogeneity of evaluation in order to make definitive assertions and meta-analyses.

## Figures and Tables

**Table 1:** Studies focused on experimental model heart tissues.

Study (Year)	Model	Methods	Altered miRNAs	Cardiotoxicity Assessment	Other Issues
Vacchi-Suzzi et al. (2012) [22]	Rats (Sprague Dawley)	8 groups of 6 male rats: (1) Control group with saline (Vehicle) (2) DOX 1 mg/kg/week, IV; (3) DOX 2 mg/kg/week, IV; (4) DOX 3 mg/kg/week, IV; (5) DEXRA 50 mg/kg, IP (alone); (6) DEXRA 50 mg/kg, IP + DOX 2 mg/kg/week, IV; (7) EPS 1 mg/kg/week, IV; (8) EPS 3 mg/kg/week, IV At 2-, 4-, and 6-weeks samples were collected	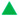 miR-34c 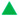 miR-208b 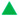 miR-215 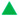 miR-216b 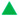 miR-367	- Vacuolation - Altered regulators: Ambra 1 Myh6 Ankrd1/Carp Nppb Sipa1	- Only 2 out of 6 mice survived at 3 kg/mg for 6 weeks - Quantification and determination of vacuolation not clear
Desai et al. (2014) [23]	Mice (B6C3F1)	5 groups of dox-treated rats at the dose 3 mg/kg (n = 12) and 5 groups of control rats (n = 10): (1) DOX for 2 weeks (c.d. 6 mg/kg) (2) DOX for 3 weeks (c.d. 9 mg/kg) (3) DOX for 4 weeks (c.d. 12 mg/kg) (4) DOX for 6 weeks (c.d. 18 mg/kg) (5) DOX for 8 weeks (c.d. 24 mg/kg)	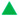 miR-34a 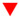 miR-150 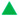 miR-208b 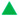 miR-21 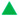 miR-221 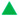 miR-222	cTnT Measurement: - Sensitivity 0.01 mg/kg - cTnT 0.026 at c.d. 18 mg/kg - cTnT 0.036 at c.d. 24 mg/kg - Cardiomyocyte vacuolization	- Mice were sacrificed after a week. Acute damage was not investigated - TnT was investigated with a human validated kit; there are concerns for reproducibility in mice.
Fu et al. (2012) [24]	Rats (Wistar Albino)	4 groups of 5 male Rats (N = 20) treated with 3 mg/kg/day of daily DOX: (1) Control group (2) Treatment days (c.d. 6 mg/kg) group, (3) Treatment days (c.d. 12 mg/kg) group, (4) Treatment days (c.d. 18 mg/kg) group	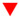 Let-7g	- Heart rate (n.c.d.) - Blood pressure (n.c.d.) - cTnT (slight increase)	- Control group FU was 6 days, and there were not control groups directly comparable for DOX 6 and 12 mg/kg c.d. - Blood pressure and heart rate not reliable as they could increase with stressing factors before the sacrifice
Tong et al. (2015) [25]	Mice (Balb/C) and H29C cell lines	4 groups of 10 male Balb/C mice each: (1) A-DOX: 4 mg/kg for 5 days (c.d. 20 mg/kg, IP) (2) A-CS: Acute saline control group (3) C-DOX: 5 mg/kg/week for 4 wks (c.d. 20 mg/kg, IP) (4) C-CS: Chronic saline control group	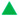 miR-21	- LVSP - LVEDP - −dP/dt - +dP/dt	- Mice for acute model were sacrificed 6 days after the last injection; this could account for a loss of information on early mechanisms and for the consequent loss of statistical significance
Tony et al. (2015) [26]	Mice (Balb/C)	3 groups of 42 female Balb/C mice each: (1) DOX 20 mg/kg (n = 23) (2) DOX 20 mg/kg + antagomir (50mmol) from day -4 (n = 8). (3) Control group, vehicle (n = 11) (4) Mice were sacrificed after 7 days from last injection.	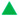 miR-208	- Echocardiography - Apoptosis assays	- Single dose was administered; thus, cardiac damage could not be reiterated - In this case, mice were sacrificed after 7 days; thus, early modifications were lost
Roca Alonso et al. (2015) [27]	Rats Sprague-Dawley	20 Sprague Dawley rats were divided in 3 groups: (1) LAD ligation coronary group (n = 10), with a surgical ligation of anterior-descendant coronary; (2) EARLY MI-induced rats, sacrificed after 4 wk (n = 5); (3) LATE HF-induced rats, sacrificed after 16–20 wk (n = 5) (4) DOX-induced HF model (n = 10), treated with DOX 15 mg/kg delivered via 6 injections in 2 wks. (5) Acute DOX in vitro model, Rat ventricular cells were treated with DOX 1 umol/L	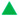 miR-29c 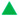 miR-30e 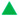 miR-133b 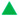 miR-143 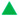 miR-210 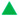 miR-345	None	- A potential involvement of GATA6 was speculated from authors, but link analysis was not performed - Cardiac assessments were not performed (cTnT or US)
Piegari (2016) [28]	Rat culture cells	Rat CPC were harvested from Fisher 344 rat hearts: (1) rCPCs DOX 0.5 μM (2) H9c2 DOX 0.5 μM (3) Fibroblasts DOX 0.5 μM (4) RAOECs DOX 0.5 μM	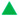 miR-34a	None	- Cardiac assessments were not performed (cTnT or US) - Lack of a vehicle group as control
Yin et al. (2016) [29]	Mice (C57BL/6)	Male C57BL/6 mice treated with: (1) DOX 25 mg/kg (2) DOX 25 mg/kg + mir-320 (3) DOX 25 mg/kg + antagomir-mir-320 (4) In vitro cardiac cells DOX 1mmol/L for 6 h as a control group	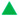 miR-320	- Echocardiography - In vivo hemodynamic - Myocyte apoptosis - Nitric oxide release	Not stated the assessed cases of heart injuries in the MI-induced group
Holmgren et al. (2016) [30]	Human culture cells	Human cardiomyocytes trteated with: (1) DOX (50 nM, 150 nM, or 450 nM) for up to 2 days (2) Control group (vehicle)	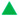 miR-34a 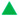 miR-34b 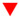 miR-146 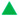 miR-187 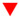 miR-15b 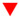 miR-199a 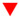 miR-214 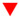 miR-130b 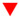 miR-424	- Cell morphology - Cell contractility	Lack of precise definition of cardiotoxicity: just defined as generic “change in morphology” and a “more unsynchronized beating”
Novak et al. (2017) [31]	Rats (Wistar)	3 groups of Wistar Rats (Ntot = 29): (1) Control group, vehicle (n = 10) (2) DOX 5mg/kg (n = 10) (3) L-DOX 5 mg/kg (n = 9) After 24 h, the animals were sacrificed	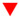 Let-7g 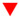 miR-208a	None	- Only acute injury setting was evaluated - Cardiac assessments were not evaluated; therefore, difficult to associate miRNa modifications with real cardiac damage in this study.

A-DOX: DOX-related acute injury model; C-CS: control saline group in chronic injury model; c.d.: Cumulative dose; cTnT: Cardiac Troponin T; DZR: Dexrazoxano; DOX: Doxorubicin; +dP/dt: maximal slope of systolic pressure increment; −dP/dt: the maximum rate of left ventricular pressure decline; EPS: Etoposide; IP: intraperitoneal; IV: intravenous; LAD: left anterior descendant coronary; L-DOX: Liposomal Doxorubicin; LVEDP: Left Ventricular End Diastolic Pressure; LVSP: Left Ventricular Sistolic Pressure; N.C.D.: not correlated with cardiac damage; rCPCs: rat Cardiac Progenitor Cells; RAOECs: Rat Aortic Endothelial Cells, Wks: weeks. 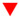 Decreased expression; 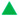 Increased expression.

**Table 2:** Studies focused on circRNAs in the Cardiotoxicity setting.

Author (Year)	Methods	Cardiac Damage Assessment	circRNA Profiles	Potential Role	Limitations
Gupta et al. (2018) [44]	- Male C57BL/6 N mice: DOX at 5 mg/kg i.p. once a week for 5 weeks. Euthanized one week later. - In vitro experiments (HL-1 cell line, NRC, HPSC) inducing QKI5 overexpression through injection of lentiviral AAVV at high doses (5 × 10^3^) and low doses (1 × 10^5^)	- Echocardiography - In vitro experiments used as validation of in vivo results	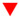 Circ-Ttn 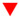 Circ-Fhod3 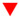 Circ-Strn3 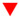 Circ-Arhgap32 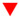 QKI (RBP)	- Diagnosis of DOX-induced heart damage through QKI/circ-Ttn/circ-Fhod3/circ-Strn3/Circ-Arhgap32 levels - Treatment of DOX-induced damage through low dose siRNA doses, overexpressing QKI family - Treatment of DOX-induced damage through low dose of QKI5 injections.	- In vivo study lacks Troponins investigation - DOX cumulative concentrations in relation with the decreasing doses of QKI5 in heart toxicity were not assessed. - The heterodimerization vs. homodimerization of QKI5, QKI6, QKI7 in order to assess cardiac damage vs. cardioprotection was not easily predictable
Ji et al. (2020) [43]	- H9c2 cells: treated with DOX 2 μM or 0.2 μM - Male C57BL/6J mice: treated twice per week with 10 mg/kg DOX or control for a week (20 mg/kg c.d. of DOX). Heart tissues were explanted a week later	- Apoptosis - Histopathology - Echocardiography	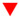 Circ-Pan3 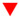 QKI (RBP)	- Circ-Pan3 is downregulated after QKI inhibition actuated by the overexpressed miR-31-5p - Circ-Pan3 and mir-31-5p could be used as biomarkers	- Troponins and pro-BNP were not investigated
Han et al. (2021) [45]	DOX treated human-induced pluripotent stem cell-derived cardiomyocytes (hiPSC-CMs) - Autopsy specimens obtained from patient with DOX-induced HF A wide expression profile was performed, screening 19112 circRNA.	- Histopathologically - LDH leakage - Cardiomyocyte - necrosis index - Number of EthD1-positive dead cells	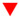 Circ-ITCH	- CircITCH act decreases the cellular and mitochondrial oxidative stress - CircITCH limits DNA damage DOX-induced - CircITCH acts also sponging miR-330-5p as a direct target. - Inhibiting mir-330-5p negatively regulated the pathway for SIRT6, BIRC5, and ATP2A2.	- Possible side effects rather than physiological mechanisms could be an overdosing effect due to the high overexpression efficiency of circRNA - The choice to use hiPSC-CMs harvested from human cells instead of validated cell lines could undermine comparability and reproducibility - Lack of clinical evidence of cardiac damage following the guidelines’ criteria (FE, cTnI, etc).
Li et al. (2021) [15]	**AC16 cell line:** cells were treated with crescent DOX concentrations (DOX, 2.5, 5, and 10μM and control (vehicle)) for 24 h. DOX 5 μM was selected as concentration for further analysis	- Cell viability assay - Cell apoptosis assay	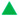 Circ-Ska3	- Potential biomarker of TLR-4 pathway activation - Cardiac damage diagnosis secondary to activation of circ-SKA3/miR-1303/TLR-4 - Biomarker modulator of mir-1303	- No studies were conducted in vivo - Small sample size of cell AC16 was used
Wang et al. (2021) [46]	12 Male C57BL/6J mice: E- xperimental group (n = 6) treated with 5 mg/kg DOX per week for 5 weeks - Control group (n = 6) treated with an equal volume of saline (0.1 mL) for 5 weeks. - Eventually mice were sacrificed and heart explanted	- Cell viability - LDH releasing - ROS production and Lipid peroxydation	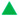 Circ-Arhgap12	- Potential biomarker for oxidative stress - Potential biomarker for apoptosis, atherosclerosis, and cardiac damage - Demonstration of CTX through miR-135a-5p inhibition	- LDH used as generic myocardionecrosis marker instead of more specific marker such as CK-MB or myoglobin. - Time of sacrifice was not mentioned. Consequently, we cannot know if acute or chronic damage was studied
Xing et al. (2021) [47]	12 male C57 mice: - Induction group (n = 6) treated with i.p. injections with DOX 15 mg/kg - Control group (n = 6) received an equal quantity of saline (0.1 mL/10 g). 5 days after the treatment, a blood sample was collected, prior to sacrifice.	- Histopathological changes of heart tissue Serum CK-MB elevation	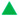 Circ_0016006 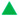 Circ_00115773 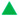 Circ_0002106	- Mmu_circ_0002106 sponges miR-344g-3p and miR-22-3p (cell growth, apoptosis, motility, and cell cycle) - Mmu_circ_0015773 sponges miR-470-5p and miR-679-5p, miR-296-3p andmiR-876-5p (cell proliferation, the cell cycle, and cell apoptosis) - Mmu_circ_0016006 sponges miR-466i-5p, miR-665-3p, and miR-466m-3p (cell apoptosis)	Biomarkers Troponins and pro-BNP were not dosed

CK-MB: Creatinkinase muscular isoform; i.p.: intraperitoneal; HF: Heart Failure; HPSC: Human Pluripotent Staminal Cardiomyocytes; pro-BNP: Pro Brain Natriuretic Peptide; NRC: Neonatal Rat Cardiomyocytes; QKI: Quaking; RBP: RNA Binding Protein. 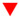 Decreased expression; 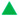 Increased expression.

**Table 3:** Studies analyzing exosomes.

Author (Year)	Methods	Cardiac Damage Assessment	Exosome Content	Potential Role	Limitations
Milano et al. (2019) [50]	Sprague-Dawley rats treated with DOX for 6 cycles for a c.d. of 15 mg/kg followed by Trastuzumab for other 6 cycles and a c.d. of 20 mg/kg Exosome isolation: Exosome purified from human cells derived from right appendage surgically removed by ultracentrifugation (gold standard)	Echocardiography was performed on Days 0 (baseline), 12, 19, 30, and 37	miR-146a -5p	Mesenchimal exosomes can restore the senescence DOX-induced through MiR-146a-5p modulating: - Traf6 - Smad4 - Irak1 - Nox4 - Mpo Mediators of inflammatory, cell death, innate immunity, sterile inflammation, cardiac dysfunction, and myocardial fibrosis in mice	- No mention if miR-146a-5p was decreased in the DOX-treated mice - The cTnI was not dosed - Lack of a clear definition of CTX - No mention if echography was performed by blinded researchers: possible exposition to performance bias
Beaumier et al. (2020) [13]	**9 canine patients** underwent to 5 cycles of IV DOX: - 30 mg/m^2^(if dogs >15 kg) - 1 mg/kg (for dogs <15 kg) For 2 or 3 weeks. Exosome purification: blood sample swere collected at 1st, 3rd cycle and 1M post-DOX, and after a centrifugation of 1320× *g* for 10 min, exosomes were isolated through IZON size exclusion chromatography (SEC)	- ECT: 1st, 2nd, 3rd, 5th cycle and 1 M after treatment completion - Blood sample before DOX treatment and 1 month after treatment completion. cTnI cut-off concentration >0.08 ng/mL - Histologic examination of the right atrium, right ventricle, interventricular septum, and left ventricle	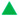 miR-181d 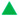 miR-502 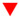 miR-107 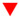 miR-146a	MiR-107, miR-146a, miR-181d, miR-502, or their combination best fitted as potential biomarkers for DOX-induced cardiotoxicity.	- Small sample size and no control arm - The cTnI increases were not statistically significant until 1 month post-DOX vs. baseline values - The cTnI did not correlate with LVEF - until 1 month after treatment completion vs. baseline values - Low rate of heart injury clinically assessed, although autopsy revealed a higher rate
Zhuang et al. (2020) [51]	**Male C57/Bl6 mice** injected with 3 cyles ip of 4 mg/kg on alternative days in a time span of 1 week For isolating exosomes, exosome quick extraction, and stored at 4 °C for at least 12 h	Echocardiography 14 days after treatment with Dox	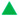 miR-221-3p	Protective roles against through SIRT-2: - Cell death - Apoptosis - Cell senescence	- The cTnI levels were not measured during treatment period - US was not declared if performed at the baseline - Signs of cardiopathy and miRNA expression profiles were not studied for correlations - Low efficient method for extracting exosomes
Sun et al. (2020) [52]	**C57Bl/6 mice** injected ip with DOX at 5 mg/kg/wk for 4 consecutive weeks Exosome isolation: preliminary centrifugation twice (2500× *g*, 4 °C, 15 min). Samples were ultrafiltrated twice and ultracentrifuged at the end.	Echocardiography	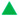 miR-21	Exosomes enriched with miR-21 inhibits cardiomyocyte apoptosis via repressing PDCD4	US was not declared if performed at the baseline. Two techniques for exosome purification were used. Optimal purification was reached, but the drawback could be a loss of exosomes in the process.
Xia et al. (2020) [53]	**hiPSCdC** were pre-treated with Hypoxia stimulus, and then they were administered with 0.5 μM for a duration of 24 h Exosome isolation: After the cells were cultured for 48 h, the supernatants were collected with the exosome quick extraction solution	Cardiomyocyte senescence measured via β-galactosidase assay	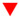 miR-92a-3p	Exogenous hypo-Exo can restore through the axis lncRNA-MALAT1/miR-92a-3p/ATG4A: - Metabolism disorders - Senescence - Growth arrest of normal somatic and postmitotic cells	- Lack of an in vivo model to confirm the results - Low efficient method for extracting exosomes
Xia et al. (2021) [54]	+hiPSCdC were pre-treated with hypoxia stimulus, and then they were administered with 0.5 μM for a duration of 24 h, and exosomes (hypo-Exo) were collected from supernatants. Male C57/Bl6 mice were injected with hypo-Exo. Exosome isolation: after the cells were cultured for 48 h, the supernatants were collected. The exosome quick extraction solution was used	- Cardiomyocyte senescence measured via β-galactosidase assay Cell proliferation assay and Cell cycle assay - Relative telomere length measurement/Relative telomerase activity measurement	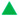 miR-199a-3p	Exo-miR-199a-3p/GATA4 axis stimulates: - Survival factors - Cell cycle regulatory - Proteins - Senescence	Low efficient method for extracting exosomes
Lee et al. (2021) [55]	H9c2 cardiac myoblast cells pre-treated with cSMC-EV harvested from mesenchymal stem cells 24 Male C57BL/6 mice were randomly assigned to: - Control (n = 8), - DOX (n = 8), - MSC-sEVs + DOX (n = 8) At 14 days, the animals were sacrifced Exosome isolation: after the cells were cultured for 24 h, the supernatants were collected. Ultrafiltration-based purification via MWCO filter was used for exosome purification	Echocardiography performed at 14 days	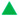 miR-199a-3p	cMSC-EV containing miR-199a-3p positively regulates: - Survivin - Akt-Sp1/p53 pathway	- No mention if echography was performed by blinded researchers: possible exposition to performance bias - CTnI was not dosed - Correlation analysis among miRNAs and echocardiographic alterations was not performed
Li et al. (2021) [15]	AC16 cells were exposed to concentrations of DOX 2.5 μM, 5 μM, and 10 μM for 24 h. Exosome isolation: after a centrifugation at 3000× *g* for 15 min, Exoquick exosome precipitation solution was used	None	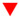 miR-1303	- Marker for early toxicity through miR-1303/ circSKA3/TLR-4 - Marker for inflammation pathway activation (TLR-4)	- Small sample size - In vivo experiments required to validate the results
Lei et al. (2021) [56]	85 Sprague-Dawley rats treated with 6 IV doses of doxorubicin (Sigma) delivered at regular intervals from day 1 to day 11 (c.d. 15 mg/kg) Exosome isolation: exosomes collected from bone marrow MSC (Exo-BMSC) on day 28 and consequently purified through ultracentrifugation (gold standard)	- Echocardiography performed on day 28 - CK-MB, cTnI, pro-BNP - Histological examination	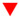 miR-96	miR-96 suppresses DOX-induced activation of upregulation of Rac-1, following the axis miR-96/Rac-1/NF-Kb after DOX treatment	- No mention if echography was performed by blinded researchers: possible exposition to performance bias

C.d.: cumulative dose; cTnI: cardiac troponin I; cSMC-EV: cardiac Stem Mesenchymal Cells exosomes; Exo-BMSC: exosomes derived from Bone Marrow Stem Cells; Hypo-Exo: Exosomes from stem cells pre-treated with hypoxia; IV: intravenous. 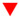 Decreased expression; 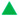 Increased expression.

**Table 4:** Studies focused on miRNAs in human blood samples described in literature.

Author (Year)	Methods	Cardiac Assessment	miRNA Profiles	Potential Role	Limitations
Rigaud et al. (2017) [35]	- 4 cycles of AC-TAX - Blood samples were collected 3 wks after each cycle	- Echocardiography (C2, C4, M6, M9) - CTnI	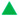 miR-1 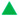 miR-133b 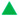 miR-146a 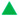 miR-423-5p	Arrhythmia, MI, CH, HF, proliferation, differentiation, survival, hypertrophic growth	None of the miRNAs evaluated showed any difference when controls were compared with the cardiotoxicity group
Todorova et al. (2017) [58]	- 20 BC pts underwent AC - Blood samples collected at baseline and after C1	Multigated acquisition (MUGA)	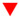 miR-1 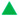 miR-126 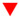 miR-210 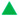 let-7f	Inflammatory response, immune trafficking, injury response, cellular growth, apoptosis, organismal development, MI, CH, HF	- Small sample size - Blood sample collection was made only at baseline and after first cycle - CTX was defined only by LVEF depression with no mention of HF (LVEF<50%)
Leger et al. (2017) [59]	- 33 patients <18 years administered with Anthracycline vs. patients administered with non-cardiotoxic agents - Blood samples were collected at 6, 12, and 24 h	- High sensitivity Troponins	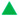 miR-1 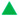 miR-29b 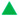 miR-499	Inhibitors of cardiac fibrosis, MI, CH, HF, hypertrophy, maladaptive remodeling, oxidative stress	- Small sample size - Heterogeneity of type of cancers and of therapeutic schemes - No cardiac diseases described, just cTn elevation - Echocardiographic alterations are late findings, and no data were collected in the chronic setting to assess predictivity of miRNA
Zhu et al., 2017 [60]	- 79 TNBC patients were selected for an EC-D neoadjuvant scheme - LVEF and blood samples were performed at baseline and C4, C8, M3, M6, M12	Cardiotoxicity was defined as clinical evidence for: (1) heart failure (2) ACS (3) fatal arrhythmia (4) LVEF decline by 10% below 53%	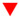 let-7f 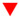 miR-126 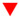 miR-19a 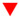 miR-20a	Pro-angiogenic role regulating angiogenic growth factors (VEGF and TGF-β), increasing oxygenation, potential role in protecting from ischemia and reducing apoptosis (Nf-Kb pathway)	- Small sample size - Monocentric study - CTX events were too few to perform the survival analysis and hazard regression analysis
Qin et al. (2018) [61]	- 365 BC patients were selected for EC-D neoadjunat scheme - LVEF and blood samples were performed at baseline and C4, C8, M3, M6, M9, M12	Cardiotoxicity was defined as clinical evidence for: (1) LVEF decline by 10% below 53% (2) ACS (3) fatal arrhythmia (4) heart failure	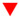 let-7f 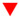 miR-17-5p 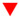 miR-20a 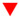 miR-126 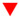 miR-210 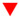 miR-378	Myocardial oxidative stress, anti-angiogenesis (VEGF signaling pathway), impairment in perfusion, impairment in tissue recovery, myocardial fibrosis	- Selection biases due to monocentric study type and enrolled only people from north China - Blood samples collections only before NACT but not at other periods - Trastuzumab positively correlated with CTX incidence causing biases for DOX-induced CTX assessment.
Oatmen et al. (2018) [62]	- Pediatric patients underwent anthracycline-based therapy - MRI and blood samples collection at 24–48 h post-infusion and at therapy completion (about 6 cycles)	- Cardiac MRI	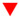 miR-181 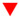 miR-199 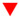 miR-107 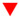 miR-499 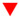 miR-145 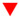 miR-100 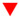 miR-103 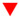 miR-142	Myocardial injury, myocardial growth, myocardial disease and dysfunction, myocardial differentiation and development	- Lack of a comparison with baselines level of miRNA (post first cycle vs. post last cycle) - Small sample size - Pediatric patients (not comparable with adults)
Frères et al. (2018) [36]	- 45 BC underwent neoadjuvant scheme with EC + TAX - Plasma collection at baseline, C2, at completion 8 days before surgery and M3	- cTnT - NT-proBNP	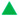 miR-34a 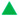 miR-126 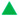 miR-199a 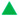 miR-423	Myocardial infarction, cardiomyocytes regeneration, recovery, induced p53 anti-tumor effect	- Small sample size - Only one cardiac disease observed - Patients treated with trastuzumab were not excluded; LVEF decline in this subset is not evaluable for Epirubicin alone
Gioffré et al. (2020) [63]	- 88 BC patients were treated with DOX (n = 32) and EPI (n = 56) - Samples were collected at baseline, at C1, C2, C3, C4 and M1, M3, M6, M12	- cTnI and CTnT - Echocardiography	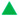 miR-122 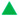 miR-499a 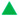 miR-885	Not evaluated	- Small Sample size - No cardiac disease or LVEF depression was reported, only cTn elevation - Different kits in different hospitals were used
Lakhani et al., 2021 [64]	- 17 TNBC patients were treated with DOX - Blood samples and echocardiogram at baseline at 3 mts and 6 mts from DOX beginning	- Echocardiography - Plasma levels cTnT, pro-BNP, MPO, TOP-2b, IL-6, MMP-2	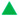 miR-29a 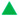 miR-34a 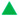 miR-126 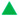 miR-423 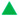 miR-499a	Cellular apoptosis, cardiac regeneration, oxidative stress, cardiac remodelling, cardiac hypertrophy, heart failure	- Not mentioned the setting of chemotherapy (metastatic, neoadjuvant?) - No mention of the chemotherapeutic associations (was Cyclophosphamide associated?)
Alves et al. (2022) [65]	6 BC patients were treated with DOX: - 4 women were Her-2+ BC - 2 women were TNBC Samples were collected at baseline and 7 days after the treatment end	- cTnI and NT-proBNP were dosed - Echocardiography - patients were selected with a moderate-high risk to develop CTX (overweight, DOX c.d.>250 mg/mq) - CTX was defined as drop of LVEF below the value of 50% or a reduction in LVEF of 10% under 50% or an elevation in Troponins of 20% from baseline	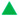 miR-133a 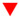 miR-133b	Cellular apoptosis cell survival, prognostic and diagnostic factors for CTX damage	- Small sample size (only 6 patients) - Chemotherapeutic regimens were not declared in methods - 2 women Her-2+ underwent trastuzumab therapy. No mention if balanced between DOX and control group. The effect of trastuzumab on ERBB2 pathway could alter the results of altered miR-133 expression.

AC: Adriamycin 60mg/mq) and Cyclophosphamide (600 mg/mq), every 3 weeks (q3w) for 4 cycles; ACS: Acute Coronary Syndrome; AC-TAX: Adriamycin and Cyclophosphamide, then sequential. 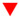 Decreased expression; 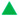 Increased expression.

## Data Availability

Not applicable.
